# Mitochondria and Lysosomes: Discovering Bonds

**DOI:** 10.3389/fcell.2017.00106

**Published:** 2017-12-07

**Authors:** Kiran Todkar, Hema S. Ilamathi, Marc Germain

**Affiliations:** ^1^Groupe de Recherche en Signalisation Cellulaire and Département de Biologie Médicale, Université du Québec à Trois-Rivières, Trois-Rivières, QC, Canada; ^2^Centre de Recherche Biomed, Université du Québec à Trois-Rivières, Trois-Rivières, QC, Canada

**Keywords:** mitochondria, lysosome, TFEB, reactive oxygen species, Ca^2+^, inter-organelle contact site

## Abstract

In the last decade, the traditional view of lysosomes has been challenged by the recognition that lysosomes are not only degradative organelles, but also metabolic sensors that play a key role in the regulation of metabolism and cell growth. Similarly, mitochondria are now seen as crucial metabolic hubs dictating cell fate decisions, not just ATP-producing machines. Importantly, these functions are generally performed as a coordinate response of distinct organelles that are physically and functionally connected. While the association between mitochondria and the endoplasmic reticulum is well known, a similar interaction between mitochondria and lysosomes is now emerging. This interaction could be required to shuttle amino acids, lipids and ions such as Ca^2+^ between the two organelles, thereby modulating their metabolic functions. In addition, a tethering complex linking the two organelles has recently been described in yeast, although the mammalian counterpart has yet to be identified. Here, we discuss the implications of these recent findings.

## Introduction

In the last decade, there have been major changes in the way we understand the function of organelles. For example, lysosomes, acidic organelles that contain an array of hydrolases required for the degradation of macromolecules, have historically been known as degradation centers. However, we now know that they also play a key role in nutrient sensing, and act as a reservoir for amino acids and ions such as Ca^2+^ (Reviewed in Appelqvist et al., 2013; Ballabio, 2016). Similarly, mitochondria, the “powerhouse of the cell,” are much more than an ATP factory. They perform a diversity of cellular roles (reviewed in Patten et al., 2010; Nunnari and Suomalainen, 2012; Mailloux et al., 2013; Raimundo, 2014), including many of the key reactions of intermediary metabolism, control of apoptosis, reactive oxygen species (ROS) signaling, and control of cellular differentiation during development (Kasahara et al., 2013; Khacho et al., 2016). A second aspect of organelle biology that has come to the fore in recent years is the presence of interorganelle contact sites that physically and functionally connect distinct organelles. While mitochondria-endoplasmic reticulum (ER) contact sites have been known for years, this has now been expanded to other organelles including lysosomes-mitochondria interactions (Elbaz and Schuldiner, 2011; Daniele and Schiaffino, 2014). Direct contact sites between mitochondria and lysosomes have been suggested to be required for lipid transfer between the two organelles (Elbaz-Alon et al., 2014; Honscher et al., 2014), although their exact role remains to be elucidated.

**Figure 1:**
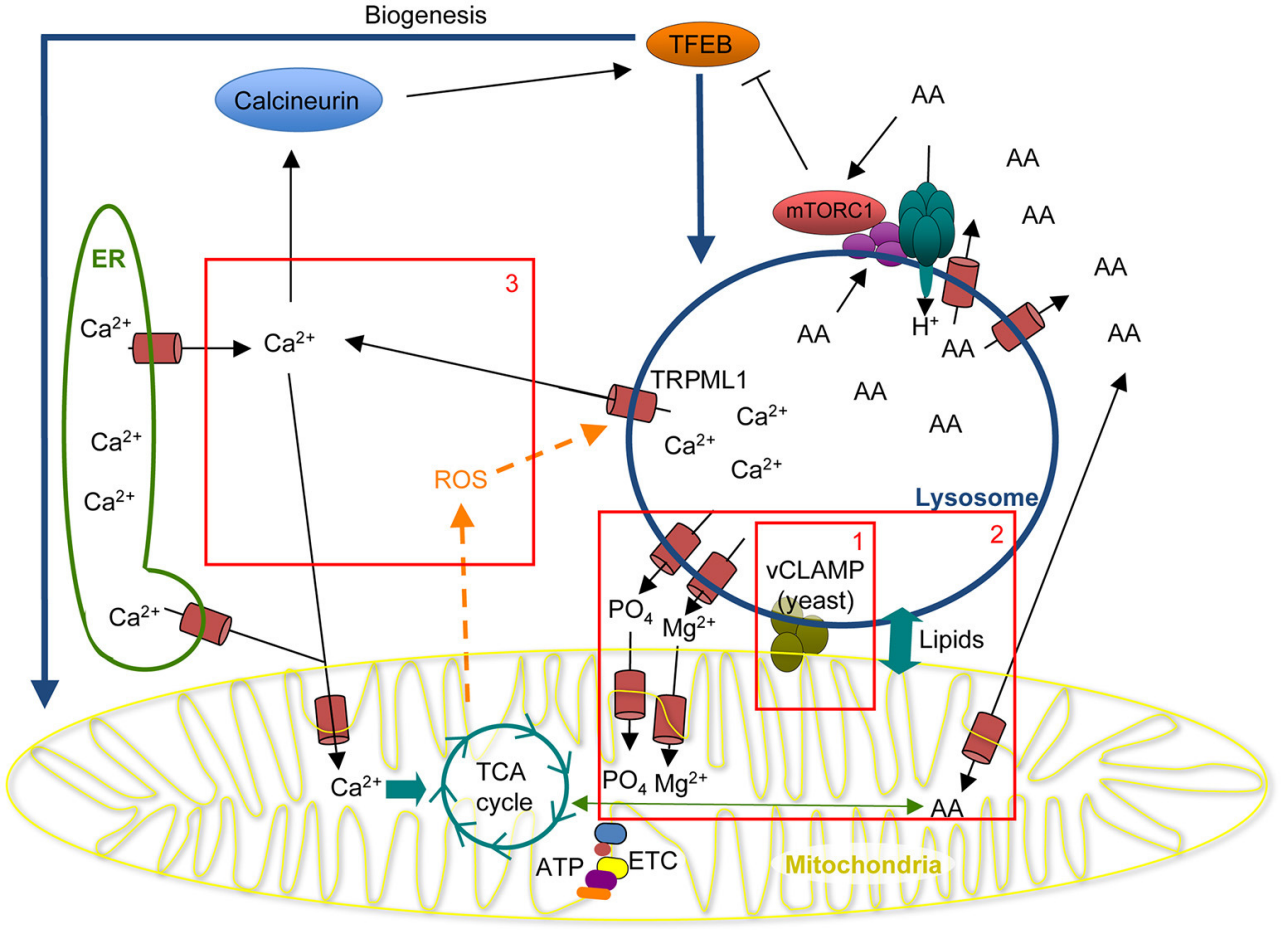
Model for the physical and functional interaction between mitochondria and lysosomes. Boxed areas represent outstanding questions: (1) The physical interaction between mitochondria and lysosomes occurs through vCLAMP in yeast but the identity of the proteins mediating this interaction in mammalian cells remain to be determined. (2) Lipid and amino acid metabolism are controlled by both lysosomes and mitochondria. However, how the interaction between the two organelles affects metabolism is still unknown. Possible mechanisms include lipid and amino acid transfer, as well as exchange of ions that regulate mitochondrial function. (3) Ca^2+^ is a key signaling molecule regulated by both lysosomes and mitochondria, in addition to the ER. Ca^2+^ activates several cellular processes including Calcineurin-dependent activation of TFEB and mitochondrial metabolism. As mitochondrial ROS stimulates lysosomal Ca^2+^ release, the role of Ca^2+^ in controlling lysosome-mitochondria cross-talk needs to be addressed. AA, amino acids; ETC, Electron transport chain; ER, Endoplasmic reticulum.

Interestingly, alterations in mitochondria and lysosomes are often present concomitantly in the neurons of patients affected by neurodegeneration, suggesting close functional links between mitochondria and lysosomes. In fact, a wide range of muscular and neurological disorders are caused by mutations in specific mitochondrial or lysosomal proteins. Many lysosomal storage diseases caused by mutations in lysosomal acid hydrolases exhibit neurodegeneration as a prominent clinical feature. Similarly, mutations in mitochondrial proteins often cause neurological diseases. These mutations directly affect the structure and function of these important organelles, leading to oxidative stress, accumulation of undigested intracellular material and impaired cellular function (reviewed in Vafai and Mootha, 2012; Appelqvist et al., 2013; Raimundo, 2014; Ballabio, 2016; Lloyd-Evans and Haslett, 2016). The functional and physical association between mitochondria and lysosomes is thus emerging as a key determinant of cell function. Although the mechanisms through which these interactions occur remain to be elucidated, we hypothesize that they are required to shuttle important metabolites and ions between the two organelles (Figure 1, outstanding questions are represented by boxes 1–3).

## Functional crosstalk between mitochondria and lysosomes

Macromolecules to be degraded reach lysosomes by two main routes: endocytosis and autophagy. During autophagy, cytoplasmic material is packaged into a double membrane vesicle (autophagosome), which then fuses with a lysosome (reviewed in Boya et al., 2013; Shen and Mizushima, 2014; Xu and Ren, 2015). In addition to starvation-induced bulk autophagy, a selective form of autophagy termed mitophagy is required to degrade damaged mitochondria. Disruption of this process is therefore thought to lead to the accumulation of dysfunctional mitochondria, oxidative stress, and cellular damage (reviewed in McWilliams and Muqit, 2017; Mouton-Liger et al., 2017; Rodolfo et al., 2017).

While mitophagy and the consequences of its disruption have been extensively studied, the consequences of mitochondrial dysfunction on lysosomal activity have only been recently reported. Two studies have demonstrated that mitochondrial activity is required to maintain lysosomal structure and function, following the chemical inhibition of the electron transport chain, as well as in *in vitro* and *in vivo* genetic models of mitochondrial dysfunction (Baixauli et al., 2015; Demers-Lamarche et al., 2016). Specifically, disruption of mitochondrial function causes the accumulation of enlarged endo-lysosomal structures, and impairs lysosomal acidification and activity (Baixauli et al., 2015; Demers-Lamarche et al., 2016). These lysosomal alterations were observed in a large array of mitochondrial mutants, including the deletion of TFAM (loss of mtDNA), OPA1 (disrupted mitochondrial fusion and cristae structure) and PINK1 (mitophagy), as well as in several cell types (neurons, fibroblasts, T cells; Baixauli et al., 2015; Demers-Lamarche et al., 2016). Interestingly, this was associated with the accumulation of disease-associated lysosomal substrates including sphingomyelin, which accumulates in some lysosomal storage diseases (Baixauli et al., 2015), as well as ubiquitinated protein aggregates, a prominent feature of neurodegeneration (Demers-Lamarche et al., 2016). A functional link between mitochondria and lysosomes is also supported by the phenotype of yeast lacking mitochondrial cardiolipin synthase (Cdr1). Cardiolipin is a crucial mitochondrial lipid required for mitochondrial function and thus, deletion of Crd1 disrupts mitochondrial activity. Importantly, Cdr1 mutants also show enlargement of their vacuoles (the yeast equivalent of lysosomes) and impaired vacuolar acidification (Chen et al., 2008), consistent with the lysosomal phenotype of mammalian cells with mitochondrial dysfunction.

The presence of a functional interaction between mitochondria and lysosomes is also supported by the observation that biogenesis of both organelles is controlled by the same transcriptional program dependent on transcription factor EB (TFEB; Figure 1). TFEB regulates lysosomal biogenesis by stimulating the expression of proteins involved in lysosomal activity and autophagy regulation (Palmieri et al., 2011). TFEB is activated by an increased need for lysosomal activity (such as during starvation), or when lysosomal function is impaired (Settembre et al., 2012). Interestingly, TFEB is also induced by mitochondrial dysfunction (TFAM deletion, Complex I chemical inhibition, Complex I subunit mutants; Baixauli et al., 2015; Fernandez-Mosquera et al., 2017) and has been reported to regulate mitochondrial biogenesis independent of peroxisome proliferator activated receptor-γ coactivator (PGC1α; Mansueto et al., 2017). Consistent with TFEB regulating mitochondrial biogenesis, loss of TFEB affects mitochondrial complex II activity, increases oxidative stress, and reduces ATP production (Mansueto et al., 2017). Altogether, these results indicate that mitochondria and lysosomes share strong functional links that could play a fundamental role in both normal physiology and pathology. Recent work suggests new clues about the nature of these links, including a direct physical interaction between the two organelles, as well as transfer of ions and metabolites, but their exact nature remains to be elucidated (Figure 1, boxes 1–3).

## Roles and mechanisms of lysosome-mitochondria interactions

While the mechanisms governing the functional crosstalk between mitochondria and lysosomes remain to be elucidated, the presence of common pathways regulated by both organelles could shed light on the physiological roles of this interaction. In fact, both organelles play a crucial role in amino acid metabolism and Ca^2+^ homeostasis, suggesting that these are key aspects of their interaction. In addition, a physical interaction between mitochondria and lysosomes has been reported in yeast (Elbaz-Alon et al., 2014; Honscher et al., 2014) and more recently in skeletal muscle (Aston et al., 2017). In yeast, this interaction is mediated by the vacuole and mitochondria patch (vCLAMP) that contains Vps39, a protein required for vacuolar transport. Whether the mammalian orthologs of Vps39 play a similar role still remains unknown (Figure 1, Box 1), but other proteins are likely involved. For example, the mitochondrial fusion protein MFN2 is required for the interaction between mitochondria and the lysosome-related organelle melanosome (Daniele et al., 2014). Interestingly, MFN2 has also been proposed to regulate ER-mitochondria contact sites (de Brito and Scorrano, 2008), raising the possibility of a three-way communication system that could be involved in Ca^2+^ signaling.

### Ion transfer between mitochondria and lysosomes

Ca^2+^ is a crucial signaling molecule with pleotropic roles ranging from vesicle exocytosis to cell death. While the major Ca^2+^ stores are located outside the cell and within the ER, mitochondria also play a key role in Ca^2+^ regulation (Raffaello et al., 2016). Ca^2+^ enters the mitochondrial matrix through a low affinity transporter, the mitochondrial calcium uniporter (MCU), the regulation of which is crucial for proper cell physiology (reviewed in Raffaello et al., 2016). Because MCU is a low affinity transporter, Ca^2+^ uptake by mitochondria is dependent on close contacts sites with the ER where Ca^2+^ levels reach the concentration required to activate the MCU (Raffaello et al., 2016). Ca^2+^ uptake by mitochondria ensures the rapid removal of cytosolic Ca^2+^ but also regulates mitochondrial bioenergetics by activating pyruvate dehydrogenase, α-ketoglutarate dehydrogenase, and isocitrate dehydrogenase, thereby stimulating the TCA cycle and ATP production (Denton, 2009). In addition, mitochondrial Ca^2+^ levels can act as crucial deciding factor for cell death by activating either apoptosis or necrosis (Rizzuto et al., 2012).

While the regulation of Ca^2+^ by mitochondria is well known, the role of lysosomes as a Ca^2+^ store has only recently been recognized. Lysosomal Ca^2+^ signaling regulates different cellular process such as autophagy (Medina et al., 2015), membrane fusion (Cao et al., 2015), exocytosis (Jaiswal et al., 2002), and cell death (Mirnikjoo et al., 2009). In addition to these roles, the close proximity of mitochondria and lysosomes suggests that Ca^2+^ release from lysosomes could also play a role in regulating mitochondrial activity. In fact, the activation of the Ca^2+^-dependent phosphatase Calcineurin by lysosomal Ca^2+^ release has been shown to activate TFEB (Figure 1). TFEB then stimulates the transcription of PGC1α and PPARα (peroxisome proliferator activated receptor α), inducing the expression of mitochondrial fatty acid β oxidation enzymes (Vega et al., 2000; Settembre et al., 2013). In addition, TFEB can promote mitochondrial respiration and ATP production in a PGC1α-independent manner in muscle (Mansueto et al., 2017). These observations suggest that lysosomal Ca^2+^ regulates mitochondrial catabolism through a TFEB-dependent transcriptional program.

In addition to TFEB-dependent mitochondrial biogenesis, lysosomal Ca^2+^ signals could have more direct impact on mitochondrial metabolism. This is suggested by the fact that the uptake of Ca^2+^ by mitochondria stimulates mitochondrial metabolism (Denton, 2009; Raffaello et al., 2016). Thus, similarly to ER-mitochondria contact sites, lysosomes could control mitochondrial activity through Ca^2+^ release at mitochondria-lysosome contact sites, either directly or as a result of ER Ca^2+^ release triggered by lysosomal Ca^2+^ release (Figure 1, Box 3). A role for ER Ca^2+^ in this process is supported by the observation that, upon stimulation, the initial lysosomal Ca^2+^ release is followed by ER-dependent cytosolic Ca^2+^ waves (Kilpatrick et al., 2013).

Ca^2+^ is released from lysosomes through two types of channels: two-pore channels (TPCs) and Transient Receptor Potential Mucolipin (TRPML/MCOLN) channels (Raffaello et al., 2016), the activity of the latter being regulated by ROS (Figure 1). Given that mitochondria are a major source of cellular ROS (Patten et al., 2010; Mailloux et al., 2013), this adds another potential level of crosstalk between the two organelles. In fact, the TRPML family member TRPML1 has recently been shown to act as ROS sensor, being activated by an increase in ROS to release lysosomal Ca^2+^ to the cytosol (Zhang et al., 2016). In this study, TRPML1-dependent Ca^2+^ release led to the activation of the Calcineurin/TFEB signaling cascade, increased autophagy and subsequent elimination of damaged mitochondria and excess ROS. Metabolic changes beyond autophagy were not investigated. Importantly, while physiological ROS levels likely play a key role in the coordinated regulation of mitochondria and lysosomes, increased ROS production is detrimental. This is evidenced by the observation that mitochondrial dysfunction impairs lysosomal structure in a ROS-dependent manner (Demers-Lamarche et al., 2016) and that loss of TRPML1 induces the accumulation of ROS, which in turn cause loss of mitochondrial membrane potential and fragmentation of mitochondria (Coblentz et al., 2014).

Iron is a second highly regulated ion associated with mitochondria and the endosomal compartment. Within mitochondria, iron is assembled into Iron-Sulfur clusters, inorganic cofactors that participate in a large array of cellular processes including the electron transport chain, metabolic conversion and protein synthesis (Braymer and Lill, 2017). To reach mitochondria, iron first enters the cell associated with transferrin and is subsequently released inside endosomes. Although the classical view is that iron then transit through a cytosolic labile iron pool before entering mitochondria, recent evidence indicates that iron is transferred from endosomes to mitochondria through a “kiss and run” interaction between iron-containing endosomes and mitochondria (Sheftel et al., 2007; Das et al., 2016). This mechanism would prevent the cytosolic accumulation of iron, which can catalyze the formation of damaging ROS.

### Metabolic regulation

A second essential role of lysosomes is the degradation of macromolecules, generating free amino acids, sugars and lipids that can be used in biosynthetic pathways or for energy production. As mitochondria are a major metabolic hub, lysosome, and mitochondria could regulate the function of each other through the production, transfer, or degradation of metabolites. In support of this hypothesis, mitochondria-lysosome contact sites participate in the transfer of phospholipids between the two organelles (Elbaz-Alon et al., 2014; Honscher et al., 2014; Figure 1, Box 2). Furthermore, respiratory growth on non-fermentable carbon sources in yeast increased ER-mitochondria contact sites at the expense of mitochondria-lysosome contacts sites (Honscher et al., 2014), indicating that these contact sites are actively involved in metabolic regulation. In addition, several small molecule transporters have been proposed to localize to vCLAMP in yeast (Elbaz-Alon et al., 2014), including a vacuolar phosphate transporter and a vacuolar magnesium channel. Their activation close to mitochondria could increase mitochondrial uptake of these ions, both of which stimulate mitochondrial activity (Figure 1, Box 2; Hackenbrock, 1966; Yamanaka et al., 2016). On the other hand, the regulation of lysosomes by mitochondria is independent of cellular ATP levels but can be affected by changes in NAD+/NADH ratio and mitochondrial ROS (Baixauli et al., 2015; Demers-Lamarche et al., 2016).

In proliferating cells, amino acids (especially glutamine/glutamate) provide an important carbon source for the TCA cycle (DeBerardinis et al., 2008). The close proximity of mitochondria and lysosomes could thus similarly provide an easy access to amino acids generated by lysosomal proteolysis, especially during starvation (Figure 1, Box 2). However, the role of lysosomes in amino acid homeostasis extends well beyond protein degradation. In fact, lysosomes serve as a platform to sense amino acid contents both outside and inside of the organelle (Bar-Peled and Sabatini, 2014; Lim and Zoncu, 2016). This nutrient-sensing machinery regulates the mammalian target of rapamycin (mTOR), a crucial kinase that acts as a hub for the control of cell growth and metabolism. In the presence of amino acids, mTOR activity stimulates protein translation and promotes cell growth, while inhibiting autophagy and suppressing TFEB activity. When amino acids become scarce, mTOR is inactivated. This relieves its inhibitory effect on autophagy and TFEB-dependent lysosomal biogenesis, thus promoting amino acid recycling (Figure 1; Settembre et al., 2012; Zhou et al., 2013). Interestingly, mTOR also regulates the efflux of essential amino acids from lysosomes. During amino acid starvation, mTOR inhibition leads to the selective sequestration of essential amino acids within lysosomes as a preservation mechanism. On the other hand, non-essential amino acids such as glutamine and glutamate are not affected by mTOR and are thus still released under starvation conditions (Abu-Remaileh et al., 2017; Wyant et al., 2017). As a result, they could potentially be imported into mitochondria and used as an energy source. In addition to this direct metabolic regulation, mTOR inhibition also relieves its inhibition of TFEB which, in turn, stimulates lysosomal biogenesis to help with the increased delivery of material to lysosomes caused by increased autophagy.

The metabolic changes caused by amino acid starvation also extend to mitochondria. Starvation promotes mitochondrial elongation and connectivity, and improves mitochondrial bioenergetics through ATP synthase assembly and changes in inner mitochondrial membrane (cristae) organization (Gomes et al., 2011; Rambold et al., 2011; Patten et al., 2014; Ouellet et al., 2017). While mitochondrial elongation is caused by the PKA-dependent inhibition of DRP1, a Dynamin related GTPase required for mitochondrial fission, other changes are likely controlled more directly by amino acids. For example, the OPA1-dependent narrowing of cristae width caused by starvation requires sensing by mitochondrial solute carriers (SLC25A family), including the glutamate/aspartate transporter AGC (Patten et al., 2014). In addition to these direct changes, the fact that TFEB stimulates mitochondrial biogenesis in addition to lysosomal biogenesis suggests that there is a coordinated metabolic program that is activated by amino acid starvation to promote cellular adaptation to metabolic stress. Interestingly, a recent study indicated that mTOR also regulates mitochondrial structure through a TFEB-independent pathway that relies on MTFP1, a mitochondrial protein promoting mitochondrial fragmentation. In nutrient-replete cells, a key role of mTOR is to repress 4eBP, an important translation inhibitor. However, during starvation, mTOR inactivation relieves this inhibition, thereby decreasing protein translation. As MTFP1 translation is sensitive to 4eBP, mTOR inhibition results in the loss of MTFP1 protein, which leads to mitochondrial elongation and branching, and promotes cell survival (Morita et al., 2017). Overall, these studies indicate that amino acid starvation co-ordinately regulates the function of mitochondria and lysosomes. This metabolic control is driven by mTOR and TFEB, also at a more direct level by the flux of amino acids and fatty acids between the two organelles.

## Conclusion

In the last decade, the realization that organelles interact in a close physical and functional manner has opened new research areas with important implications for our understanding of several diseases. Recent findings highlighting the physical and functional interaction between mitochondria and lysosomes suggest that this crosstalk plays a major role in metabolic regulation. However, several key questions remain unanswered (Figure 1, boxes 1–3). First, the nature of the physical interaction between the two organelles in mammalian cells remains unknown, making it difficult to assess to which extent their functional interaction requires direct physical contact. Second, both mitochondria and lysosomes have independently been studied for their role in the regulation of amino acids and lipids, but how these processes are coordinated and integrated remains an open question. Third, while both mitochondria and lysosomes are now recognized as important for Ca^2+^ regulation, how this participates to the crosstalk between the two organelles remains to be determined. Given the intimate links between mitochondria and lysosomes in disease, especially neurodegenerative diseases, these are important areas that remain to be explored.

## Author contributions

All authors listed have made a substantial, direct and intellectual contribution to the work, and approved it for publication.

### Conflict of interest statement

The authors declare that the research was conducted in the absence of any commercial or financial relationships that could be construed as a potential conflict of interest.
